# Multicentric Evaluation of SeeGene Allplex Real-Time PCR Assays Targeting 28 Bacterial, Microsporidal and Parasitic Nucleic Acid Sequences in Human Stool Samples

**DOI:** 10.3390/diagnostics12041007

**Published:** 2022-04-16

**Authors:** Felix Weinreich, Andreas Hahn, Kirsten Alexandra Eberhardt, Simone Kann, Thomas Köller, Philipp Warnke, Susann Dupke, Denise Dekker, Jürgen May, Hagen Frickmann, Ulrike Loderstädt

**Affiliations:** 1 Department of Microbiology and Hospital Hygiene, Bundeswehr Hospital Hamburg, 20359 Hamburg, Germany; felixweinreich@bundeswehr.org (F.W.); frickmann@bnitm.de (H.F.); 2 Institute for Medical Microbiology, Virology and Hygiene, University Medicine Rostock, 18057 Rostock, Germany; andreas.hahn@uni-rostock.de (A.H.); thomas.koeller@med.uni-rostock.de (T.K.); philipp.warnke@med.uni-rostock.de (P.W.); 3 Department of Tropical Medicine, Bernhard Nocht Institute for Tropical Medicine Hamburg & I. Department of Medicine, University Medical Center Hamburg-Eppendorf, 20359 Hamburg, Germany; k.eberhardt@bnitm.de; 4 Medical Mission Institute, 97074 Würzburg, Germany; simone_kann@hotmail.com; 5 Centre for Biological Threats and Special Pathogens/Highly Pathogenic Microorganisms, Robert Koch Institute, 13353 Berlin, Germany; dupkes@rki.de; 6 Infectious Disease Epidemiology Department, Bernhard Nocht Institute for Tropical Medicine Hamburg, 20259 Hamburg, Germany; dekker@bnitm.de (D.D.); may@bnitm.de (J.M.); 7 German Center for Infection Research (DZIF), Partner Site Hamburg-Lübeck-Borstel-Riems, 38124 Braunschweig, Germany; 8 University Medical Center Hamburg-Eppendorf (UKE), Tropical Medicine II, 20251 Hamburg, Germany; 9 Department of Hospital Hygiene & Infectious Diseases, University Medicine Göttingen, 37075 Göttingen, Germany

**Keywords:** test evaluation, real-time PCR, parasite, bacteria, enteric pathogen, gastrointestinal infection, diagnosis, test comparison, SeeGene, Allplex

## Abstract

Prior to the implementation of new diagnostic techniques, a thorough evaluation is mandatory in order to ensure diagnostic reliability. If positive samples are scarcely available, however, such evaluations can be difficult to perform. Here, we evaluated four SeeGene Allplex real-time PCR assays amplifying a total of 28 bacteria, microsporidal and parasitic nucleic acid sequence targets in human stool samples in a multicentric approach. In the assessments with strongly positive samples, sensitivity values ranging between 13% and 100% were recorded for bacteria, between 0% and 100% for protozoa and between 7% and 100% for helminths and microsporidia; for the weakly positive samples, the recorded sensitivity values for bacteria ranged from 0% to 100%; for protozoa, from 0% to 40%; and for helminths and microsporidia, from 0% to 53%. For bacteria, the recorded specificity was in the range between 87% and 100%, while a specificity of 100% was recorded for all assessed PCRs targeting parasites and microsporidia. The intra- and inter-assay variations were generally low. Specifically for some helminth species, the sensitivity could be drastically increased by applying manual nucleic acid extraction instead of the manufacturer-recommended automatic procedure, while such effects were less obvious for the bacteria and protozoa. In summary, the testing with the chosen positive control samples showed varying degrees of discordance between the evaluated Allplex assays and the applied in-house reference assays associated with higher cycle threshold values in the Allplex assays, suggesting that samples with very low pathogen densities might be missed. As the targeted species can occur as harmless colonizers in the gut of individuals in high-endemicity settings as well, future studies should aim at assessing the clinical relevance of the latter hint.

## 1. Introduction

As indicated by multicentric assessments [1,2] and personal experiences with samples from German soldiers, policemen and civilians returning from the tropics [3,4,5], gastrointestinal infection or colonization of the gut with pathogenic bacteria, protozoa or helminths is quite common. Consequently, molecular screening for pathogenic bacteria and parasites in stool samples for German soldiers returning from tropical deployments was established in a stepwise manner based on in-house real-time PCR assays, starting with enteroinvasive bacteria [6,7] and enteropathogenic protozoa [8,9], with a subsequent broadening of the panel by enteric helminths [10]. Although those in-house real-time PCR panels have been successfully applied even under tropical deployment conditions [11], the switch to a CE-IVD (Conformité Européenne—in vitro diagnostic) certified commercial assay has to be considered in response to Regulation (EU) 2017/746 on in vitro diagnostic medical devices (“CE-IVD Directive”), which was released in order to facilitate the standardization of diagnostic accuracy in laboratories within the European Union. In line with this regulation, in-house assays shall only be used by accredited institutes if an additional benefit compared to the available standardized commercial CE-IVD systems can be proven.

In order to comply with the CE-IVD Directive, the tropical infections laboratory of the Department of Microbiology and Hospital Hygiene, Bundeswehr Hospital Hamburg, Germany, identified the SeeGene (Seoul, South Korea) assays Allplex GI Panels 2–4 and Allplex GI Helminth(I) as a potentially suitable CE-IVD-labeled real-time PCR platform for a comprehensive screening of stool samples obtained from military returnees after tropical deployment. However, even in the case of an intended application of CE-IVD-certified commercial diagnostic assays, German diagnostic laboratories are in charge of comparative harmonization testing in order to get familiar with the accuracy of newly implemented assays, as demanded by RiLiBaek (Guideline of the German Medical Association on Quality Assurance in Medical Laboratory Examinations/“Richtlinie der Bundesärztekammer zur Qualitätssicherung laboratoriumsmedizinischer Untersuchungen”) [12]. Rabenau and colleagues published recommendations on how such laboratory-based evaluations should be performed [13,14]. Next to the assessment of sensitivity and specificity, they comprise intra- and inter-assay variations and, if applicable, the control of matrix effects, of the limits of detection and of linearity, as well as the comparison with alternative diagnostic techniques.

Prior to the switch from in-house assays to CE-IVD-labeled commercial diagnostic kits at the tropical infections laboratory of the Department of Microbiology and Hospital Hygiene, Bundeswehr Hospital Hamburg, Germany, for returnee screenings, a comprehensive assay evaluation in line with the requirements of RiLiBaek [12], as well as with the suggestions by Rabenau and colleagues [13,14], was conducted, in addition to a literature search. From our previous studies [9,10] and others [15,16,17], the initial evidence suggested that the diagnostic accuracy of the Allplex multiplex real-time PCR assays targeting bacteria and parasites in the stool samples is in a similar range as the diagnostic accuracy of the previously applied in-house assays. In line with this, successful application in tropical epidemiological studies has been demonstrated [18]. In previous studies by our group [9,10], however, residual nucleic acid extractions were used, which were prepared with other nucleic acid extraction kits than those recommended by the manufacturer. Since nucleic acid extraction is, however, an element of the CE-IVD-labeled diagnostic flow, a follow-up validation including the recommended nucleic acid extraction procedures was considered necessary by us.

The SeeGene assays Allplex GI Panel 2–4 and Allplex GI Helminth(I) cover a broad spectrum of, in part, rarely occurring bacterial, protozoan and helminth gastrointestinal pathogens, including microorganisms that rarely occur in stools samples of returnees from tropical journeys. In order to identify the positive sample materials for an evaluation, a consortium was formed whose partners contributed residual sample materials from tropical studies or diagnostic assessments of travel returnees. For selected rare pathogens, inactivated culture materials were used for the spiking experiments. Based on those samples, an evaluation of the Allplex assays in line with the suggestions by Rabenau and colleagues [13,14], including all the tested parameters, was conducted. A multicentric in-house evaluation of the performance characteristics of the Allplex assays in accordance with those evaluation standards [13,14] prior to diagnostic use was the objective of the study. As such, comprehensive evaluations are scarcely available in the literature, and the results are provided to counsel the interpretation of the diagnostic results obtained with those assays.

## 2. Materials and Methods

### 2.1. Reference Materials for the Test Evaluations

#### 2.1.1. Reference Materials Characterized by in-House Real-Time PCR

Residual samples for the test evaluation were obtained in a multicentric approach from the German military and police forces returning from the tropics [3,4], as well as from previous studies for which the diagnostic work-up was performed at our institution [19,20,21,22,23,24,25,26,27,28,29,30]. To define the strongly positive, weakly positive and negative samples, nucleic acid extractions from stool samples performed with the QiaAMP DNA stool mini kit (Qiagen, Hilden, Germany), as described by the manufacturer and elsewhere [31], were subjected to well-characterized in-house real-time PCR, as described recently [5,6,7,8,9,10,26,32,33]. Those real-time PCR protocols covered most of the target organisms of the evaluated SeeGene assays Allplex GI Panels 2–4 and Allplex GI Helminth(I), with the exception of seven parameters: *Escherichia coli* O157, hypervirulent *Clostridioides difficile*, Shiga toxin-producing *Escherichia coli* (STEC), *Aeromonas* spp., *Clostridioides difficile* toxin B, *Vibrio* spp. and *Blastocystis hominis*. Of note, the applied in-house PCR was specific for *Campylobacter jejuni* [5], while the corresponding Allplex PCR assay was generic for *Campylobacter* spp. In contrast, the in-house assay targeted *Yersinia* spp. [5], while the Allplex PCR was specific for *Y. enterocolitica*. To address this latter issue, samples were used that had also been identified as positive for *Y. enterocolitica* in previous studies using commercial PCR assays [6] or culture-based approaches [5]. Basically, it was aspired to perform the assessments with residual stool samples by applying the manufacturer-recommended mode of nucleic acid extraction using the Starlet extraction automate (SeeGene). Only in cases in which sufficient residual stool volumes were not available, residual nucleic extractions that had been prepared with the QiaAMP DNA stool mini kit (Qiagen) were used. Those individual cases are explicitly indicated in the tables of the Results section. The cut-off for the discrimination of strongly and weakly positive samples was defined at a cycle threshold value of 30 in the molecular pre-characterization real-time PCR runs, with the exception of the pathogens *E. histolytica*, *N. americanus* and *Taenia* spp., for which the cut-off had to be set at 35 due to lack of availability of samples with low Ct values.

#### 2.1.2. Reference Material Characterized by Microscopy

For the evaluation of the Allplex *Blastocystis* spp.-specific PCR assay, residual stool samples, for which microscopically positive results were shown previously [19], were applied as strongly positive samples by definition.

#### 2.1.3. Reference Materials from External Control Schemes for German Laboratories (“Ring Trials”/“Ringversuche”)

For the parameters *Escherichia coli* (STEC), *Clostridioides difficile* toxin B and *Escherichia coli* O157, predefined samples from external control schemes for German laboratories (“ring trials”/“Ringversuche”) were applied as described recently [6]. The cut-off for the discrimination of strongly and weakly positive samples for the “ring trial”/“Ringversuche” sample materials was set at 10^4^ copy numbers.

#### 2.1.4. Bacterial Suspensions Used for Spiking Experiments

Suspensions of isolates from reference collections, semi-quantified with defined Ct values of Ct 20 in the master suspension for hypervirulent *Clostridioides difficile* and *Aeromonas* spp., were purchased for the spiking experiments from microBIOMix GmbH (Regensburg, Germany). For those *Aeromonas* spp.- and hypervirulent *C. difficile*-containing suspensions for which only Ct-based semi-quantification was available, a 10-fold dilution series was established for spiking purposes, and stool samples were spiked in a 1:10 relation of pathogen-containing suspensions and stool materials each.

Further, suspensions of defined copy numbers of *Vibrio* spp. provided by the Centre for Biological Threats and Special Pathogens/Highly pathogenic microorganisms, Robert Koch Institute (Berlin, Germany), which included preparations containing DNA of the species *V. cholerae* (n = 4), *V. vulnificus* (n = 1), *V. parahaemolyticus* (n = 1), *V. fluvialis* (n = 1), *V. furnissii* (n = 1), *V. mimicus* (n = 1) and *V. alginolyticus* (n = 1), were used. Similar to the assessments with the “ring trial”/“Ringversuche” samples, stool samples spiked with >10^4^ copies of *Vibrio* spp. were prepared as strongly positive samples, with stool samples spiked with <10^4^ copies as weakly positive samples.

#### 2.1.5. Negative Control Samples for the Test Evaluations

Negative samples for the assay evaluation were characterized by the in-house real-time PCRs mentioned above. Some PCR targets of the assessed commercial real-time PCR assays were either not at all or at least not fully covered by the in-house PCR-based sample characterization. This applied to *Escherichia coli* O157, hypervirulent *Clostridioides difficile*, Shiga toxin-producing *Escherichia coli* (STEC), *Aeromonas* spp., *Clostridioides difficile* toxin B, *Vibrio* spp. and *Campylobacter* spp. (as only *C. jejuni* was covered by the in-house assay [5]). For these parameters, residual volumes from well-characterized “ring trial”/“Ringversuche” samples or samples from routine screenings of soldiers without reported external deployments or comparable risk factors for pathogen acquisition [8] were used as negative controls. This selection was made to reduce the risk of the negative control samples of being positive for the target microorganisms by chance.

### 2.2. Procedure of the Test Evaluation

The strongly positive, weakly positive and negative samples, as defined above, were subjected to real-time PCR by applying the SeeGene assays Allplex GI Panels 2–4 and Allplex GI Helminth(I) on a Bio-Rad cycler CFX 96 (Bio-Rad Laboratories Inc., Hercules, CA, USA) according to the manufacturer’s instructions. If residual stool samples or spiked stool samples and not just nucleic acid extractions were available, the stool samples were extracted by the manufacturer-recommended procedure by applying the STARMag 96 × 4 Universal Cartridge kit (SeeGene) on a Starlet extraction automate (SeeGene). Pretreatment of the samples was performed in line with the manufacturer’s recommendations. In short, stool volumes weighing 100 mg were suspended in 1 mL ASL buffer (Qiagen, Hilden, Germany). The suspension was pulse-vortexed for 1 min and incubated for 10 additional minutes at room temperature afterwards. Then, the sample was centrifuged at 20,000× *g* for 2 min in order to obtain 800 µL of supernatant for the automated nucleic acid extraction procedure. The PCR results were interpreted in accordance with the suggestions by Rabenau and colleagues [13,14] by calculating the sensitivity for 15 strongly and 15 weakly positive samples each and the specificity for 15 negative samples each, as well as the inter- and intra-assay variations, describing the concordance of 3 assessments each of the same positive sample in 3 different runs, as well as in the same run with the SeeGene platform, respectively. In cases in which sufficient numbers of positive samples could not be obtained in spite of the multicentric design of the study, deviations from the recommended study design [13,14] had to be accepted. To assess the potential influence of the extraction procedure, the obtained Ct values with the automated Starlet extraction approach and with the manual Qiagen extraction approach, as described above, were compared for samples with sufficient residual material for both nucleic acid extraction methods.

### 2.3. Ethical Clearance

Ethical clearance for an anonymized use of the residual sample materials without requirement of informed consent was provided by the medical association of Hamburg, Germany (reference number: WF-011/19, obtained on 11 March 2019). The study was conducted in line with the Declaration of Helsinki and its amendments.

## 3. Results

### 3.1. Assessment with Strongly Positive Samples

For the samples defined as strongly positive, sensitivity values ranging between 13% and 100% were recorded for bacteria and between 0% and 100% for protozoa, as well as between 7% and 100% for helminths and microsporidia. The details are provided in Table 1 below. Among the bacteria, a perfect sensitivity of 100% was recorded for the pathogens *Escherichia coli* O157, *Clostridioides difficile* toxin B and *Yersinia enterocolitica*. In the order of declining sensitivity, the bacterial targets Shiga toxin-producing *Escherichia coli*, *Campylobacter* spp., enterotoxigenic *Escherichia coli*, *Shigella* spp./enteroinvasive *Escherichia coli*, enteroaggregative *Escherichia coli*, enteropathogenic *Escherichia coli*, *Vibrio* spp. and *Salmonella* spp. followed. Less than 15 strongly positive samples containing DNA of *Escherichia coli* O157, *Clostridioides difficile* toxin B and *Yersinia enteroclitica* were available for inclusion in the study. For the two assessed decadic logarithmic dilution series comprising one inactivated strain each, four decadic logarithmic steps were tested positive for hypervirulent *C. difficile* and six decadic logarithmic steps for *Aeromonas* spp., respectively. The assessments for *Yersinia enterocolitica* were based on manually extracted sample materials only, and the assessments for *Vibrio* spp. just on the spiked samples. Focusing on protozoan parasites, 100% sensitivity was only recorded for *Dientamoeba fragilis* and the microscopically positive samples containing *Blastocystis hominis*. *Giardia duodenalis*, *Cryptosporidium* spp. and *Entamoeba histolytica* followed in declining order of sensitivity, while *Cyclospora cayetanensis* was not detected at all. For *G. duodenalis* and *E. histolytica*, less than 15 strongly positive samples were available for the study. Focusing on the helminths and microsporidia, a sensitivity of 100% was exclusively recorded for the *Ancylostoma* spp.; however, this assessment was just based on two available strongly positive samples from which only manually extracted DNA was available. While two-thirds of the microsporidia-positive samples were positively detected by the Allplex assay, the declining order of the sensitivity of helminth detections comprised *Hymenolepis nana*, *Ascaris* spp., *Taenia* spp., *Necator americanus*, *Enterobius vermicularis*, *Strongyloides* spp. and *Trichuris trichiura*. For *Enterobius vermicularis*, only six strongly positive samples could be included, and the single sample that tested positive in the Allplex assay was manually extracted. Additionally, if only samples extracted automatically in line with the recommendations for the Allplex assay were included, the proportion of positive tested samples containing *Hymenolepis nana* DNA would drop from 60% (9/15) to 45.5% (5/11). For all parameters for which the cycle threshold (Ct values) from the in-house PCR tests were available, those in-house Ct values were lower than the Ct values recorded with the Allplex assays (Table 1).

### 3.2. Assessment with Weakly Positive Samples

For the assessments with the weakly positive samples, the sensitivity of the PCR tests for bacteria ranged from 0% to 100%; of the PCR tests for protozoa, from 0% to 40%; and of the PCR tests for helminths and microsporidia, from 0% to 53%, respectively (Table 2). Among the assessments with PCR assays targeting bacteria, 100% sensitivity was recorded for residual samples containing *Escherichia coli* O157, *Clostridioides difficile* toxin B, Shiga toxin-producing *Escherichia coli* and *Yersinia enterocolitica*. In declining order of sensitivity, *Campylobacter* spp., *Vibrio* spp., enteroaggregative *Escherichia coli*, enteropathogenic *Escherichia coli*, enterotoxigenic *E. coli* and *Shigella* spp./enteroinvasive *E. coli* were also recorded, while no weakly positive sample containing *Salmonella* spp. DNA was detected. Less than 15 weakly positive samples were available for *Escherichia coli* O157, *Clostridioides difficile* toxin B, Shiga toxin-producing *Escherichia coli* and *Yersinia enteroclitica* in this study. Focusing on the protozoa, the PCR for *Dientamoeba fragilis* scored best regarding its sensitivity, followed by *Cryptosporidium* spp., while no DNA of *Giardia duodenalis*, *Cyclospora cayetanensis* or *Entamoeba histolytica* was recorded in the weakly positive samples. Focusing on helminths and microsporidia, only about half of the *Hymenolepis nana*-positive samples and a single *Trichuris trichiura*-positive sample were detected by the Allplex assay. If, however, only automatically extracted samples according to the recommendations by the manufacturer were applied, the sensitivity for *Hymenolepis nana* would have dropped from 53% (8/15) to 0% (0/6). For *Enterobius vermicularis*, less than 15 weakly positive samples were available, and half of the assessed samples could only be included as manually extracted specimens. With the exemption of *Yersinia enterocolitica*, the recorded Ct values in the in-house assays were lower than in the Allplex assays.

### 3.3. Assessments with Negative Samples

With the negative control samples as defined in the Methods section, a specificity of 100% was recorded for most of the parameters, with a few exemptions comprising bacterial targets only. A reduced specificity of 87% (2/15) was detected for enteroaggregative *Escherichia coli* (Ct 31.4, Ct 39.0), enteropathogenic *Escherichia coli* (Ct 29.6, Ct 38.6) and enterotoxigenic *Escherichia coli* (Ct 40.5, Ct 41.4), respectively, along with a reduced sensitivity of 93% for *Escherichia coli* O157 (Ct 40.4) and *Aeromonas* spp. (Ct 44.0).

### 3.4. Intra- and Inter-Assay Variation with the Assessed Allplex Assays

Very low standard deviations and the resulting variance values were calculated in the intra- and inter-assay variation assessments for the measured cycle threshold (Ct) values. In all but one instance, the recorded variances were smaller than 1. A single out-stander was recorded with a sample positive for *S. stercoralis*, for which a variance of 7.29 was calculated in the inter-assay variation assessment. The details are provided in Table 3.

### 3.5. Comparison of the Allplex PCR Ct Values after Automated and Manual Nucleic Acid Extractions

To assess the influence of the applied nucleic acid extraction approach on the results of the Allplex PCR assays, the manufacturer-recommended automated SeeGene nucleic acid extraction assay was compared to the manual nucleic acid extraction used for the in-house PCR assays (Table 4). Focusing on the bacteria, proportions of positively detected samples were higher after automated nucleic acid extraction for *Escherichia coli* O157 and Shiga toxin-producing *Escherichia coli*, identical for *Campylobacter* spp. and *Clostridioides difficile* toxin B and lower for enteroaggregative *Escherichia coli*, enteropathogenic *Escherichia coli*, enterotoxigenic *Escherichia coli*, *Salmonella* spp. and *Shigella* spp./enteroinvasive *Escherichia coli*. The maximum difference in the number of detected samples was eight. In declining order, such differences were observed for Shiga toxin-producing *Escherichia coli* (n = 8), enteroaggregative *Escherichia coli* (n = 6), *Shigella* spp./enteroinvasive *Escherichia coli* (n = 6), *Escherichia coli* O157 (n = 4), enteropathogenic *Escherichia coli* (n = 3), enterotoxigenic *Escherichia coli* (n = 2) and *Salmonella* spp. (n = 1). Lower Ct values were recorded after automated extraction for the parameters enteroaggregative *Escherichia coli*, *Escherichia coli* O157, Shiga toxin-producing *Escherichia coli* and *Clostridioides difficile* toxin B compared to the manual extraction, with higher Ct values for *Campylobacter* spp. and enteropathogenic *Escherichia coli*. Focusing on the protozoa, no difference regarding the nucleic acid extraction strategy was recorded for the *Blastocystis* spp. For all the other assessed protozoa, more positive samples were detected after manual nucleic acid extraction, but the absolute numbers of these differences were low, with n = 3 for *Cryptosporidium* spp., n = 2 for *Cyclospora cayetanensis* and *Dientamoeba fragilis,* as well as n = 1 for *Giardia duodenalis* and *Entamoeba histolytica*. Slightly higher Ct values after automated nucleic acid extraction compared to manual nucleic acid extraction could be demonstrated for *Giardia duodanalis*, *Cryptosporidium* spp. and *Dientamoeba fragilis*. Focusing on microsporidia and helminths, increased proportions of positive samples were detected after manual nucleic acid extraction compared to automated nucleic acid extraction with the Allplex PCR assays. The effect strength, however, was quite heterogenous. In declining order, increased proportions of positive detection after manual compared to automatic nucleic acid extraction were observed for *Trichuris trichiura* (difference of 86.6%), *Ascaris* spp. (difference of 73.3%), *Necator americanus* (difference of 60.0%), *Hymenolepis nana* (difference of 40.6%), *Enterobius vermicularis* (difference of 21.4%), *Taenia* spp. (difference of 10.8%), *Strongyloides* spp. (difference of 10.0%) and microsporidia (difference of 4.6%). Interestingly, significant differences between the Ct value pairs of samples positive after manual and automated nucleic acid extraction were not observed for helminths and microsporidia (Table 4).

## 4. Discussion

While molecular diagnostic strategies for the detection of bacteria, protozoa, microsporidia and helminths in human stool samples are considered well-established [34,35], the standardization and quality control for the molecular diagnosis of parasites in human stool in particular are still in the process of optimization [36,37,38]. This study was performed to evaluate the diagnostic performance characteristics of the Allplex PCR assays for the diagnosis of enteropathogenic bacteria, protozoa, microsporidia and helminths from stool samples. The study amended previous assessments that did not include the manufacturer-recommended nucleic acid extraction strategy [9,10], a strategy that deviated from the requirements of Regulation (EU) 2017/746.

The observed results were heterogenous. First, very good to excellent specificity was recorded for all the assessed parameters, in line with the previous results [9,10]. Individual PCR signals in assumedly negative samples were either observed for parameters like *Aeromonas* spp. and *Escherichia coli* O157, for which no preassessment with in-house assays was performed, or for very frequent parameters such as enteropathogenic, enterotoxigenic and enteroaggregative *Escherichia coli*, for which slightly imperfect concordance between different PCR assays is known from previous assessments [32]. Insofar, it remains unresolved whether the apparently “nonspecific” reactions were really nonspecific or just indicated truly positive samples that went undetected during the pre-characterization of the samples. The latter option was supported by the partly very high Ct values of the additional detections with the Allplex assays.

Another favorable outcome of the evaluation study was the generally low intra- and inter-assay variation of the Allplex PCR assays, as indicated by the low standard deviations and variances of the measured Ct values. Unfortunately, the remaining residual sample volumes did not allow respective assessments of both strongly and weakly positive samples. Further, for some PCR targets, the automated nucleic acid extraction could not be evaluated due to insufficient residual stool sample volumes, and so, the variation analyses had to be performed with manually extracted nucleic acids instead. In spite of these restrictions, a favorable trend was nevertheless obvious.

For nearly all the parameters, however, a lower sensitivity associated with higher cycle threshold values was recorded compared to the applied in-house reference PCRs. This phenomenon was more pronounced for parasites than for bacteria, and also, weakly positive samples were more severely affected than strongly positive ones. However, even some bacterial genera like *Salmonella* spp. were poorly detected by the Allplex assay compared to the in-house competitor assay. Hypothetically, this phenomenon might also result from a reduced specificity of the applied in-house *Salmonella* spp. PCR, but several years of successfully passed participation in external laboratory control schemes (“ring trials”/“Ringversuche”), as well as a much better concordance in a previous PCR comparison [7], do not make this explanation very likely. As a side effect of the study, it could be shown that the *Vibrio* spp. PCR missed a few of the selected *Vibrio* species included in the study, so it is unlikely to be generic for all the *Vibrio* species potentially isolated from human patients.

Interestingly, the comparably low sensitivity of the Allplex PCR assays, as observed in this study, was in contrast to previous studies comparatively targeting parasites in non-selected human stool samples [9,10]. In those studies, comparably late Ct values were also recorded for the Allplex assays; however, their overall sensitivity was not unambiguously reduced compared to the in-house competitor assays. However, partly low numbers of positive samples led to broad 95% confidence intervals in those previous studies with unselected samples [9,10] and the incomplete concordance of positive PCR results varied considerably over the different parameters. Accordingly, incomplete concordance between different PCR assays might have at least partially contributed to the apparently low sensitivity values observed in the present study with the preselected samples.

The interpretation of the results is also challenged by the fact that the target genes are not published for the Allplex assays. For coccidian protozoa like *Cryptosporidium* spp. and *Cyclospora cayetanensis*, as well as for microsporidia, previous assessments suggested considerable influence of the choice of the target gene, as well as of the number of target gene copies in the pathogen genome on the sensitivity of the diagnostic PCR assays [26,27,39]. For *Cyclospora cayetanensis*, this finding was particularly pronounced [39] and might at least partially have contributed to the recorded comparably low sensitivity of the assessed Allplex assay.

Since the observed loss of sensitivity due to the use of the Allplex assays was less striking with the manually extracted samples in the previous assessments [9,10], remaining residual sample material was used to compare the results of the Allplex assays using manually extracted DNA eluates, with the eluates prepared with the manufacturer-recommended automatic extraction procedure. Interestingly, the results were different for the various kinds of investigated pathogens. For bacteria, heterogenous results were seen for the two assessed nucleic acid extraction procedures. Both procedures showed better results with selected species regarding both the number of positively tested samples and measured Ct values. For the protozoa, slightly more detections associated with slightly lower Ct values were recorded for the manually prepared eluates, but these observed differences hardly explained the differences compared to the results of the in-house PCR assays. For helminths, strikingly more pathogen detections were seen after manual nucleic acid extraction in a species-dependent manner. Species with particular hard egg shells such as *Ascaris* spp. and *Trichuris trichiura* were among the particularly affected PCR targets but, also, species with less robust eggs like hookworms, *Hymenolepis nana* and *Enterobius vermicularis*. This more species-dependent than pathogen group-dependent effect of different nucleic acid extraction schemes on the sensitivity of helminth PCRs confirms previous findings by our group [40], while other authors have generally recommended harsh nucleic acid extraction procedures for the molecular detection of helminth pathogens [41,42,43] and even for the molecular diagnosis of protozoa [44,45]. In contrast, however, there were no significant differences regarding the Ct values of the samples, which were positive after both types of nucleic acid extraction in Allplex PCRs targeting helminths and microsporidia. Although this lack of significance may be partially blamed on the low total number of samples positive after both extractions, it at least suggests only a minor effect hardly explaining the high numbers of missed samples after automated nucleic acid extraction alone. Another phenomenon might interfere regarding this issue: As pronounced by the distributors of laboratory control schemes for helminth PCRs, optimal homogenization of the sample may be a critical factor in order to reduce the risk of nonreproducible test results due to the uneven distribution of pathogen DNA within individual stool samples [37,38]. In our study, no elaborated homogenization process for the included stool samples was performed, which might have contributed to the partially strong discrepancy in the helminth PCR results after both kinds of nucleic extraction. Therefore, it is likely that the required standardization of the pathogen enrichment as described for the microscopic detection of parasites [46] may also apply to parasite PCR. Since the in-label use of the Allplex assays in line with Regulation (EU) 2017/746 on in vitro diagnostic medical devices requires their application with the manufacturer-recommended extraction protocols in order to avoid the status of a laboratory-developed test, little can be done by diagnosing laboratories in Europe to improve the nucleic acid extraction quality by themselves. Accordingly, it would be desirable if the test producers would further address the nucleic acid extraction issue, either by further optimizing the own extraction assays for nucleic acids of the tested parasites or by broadening the in-label use of their test kits by allowing the combination with CE-IVD-labeled harsher nucleic acid extraction schemes provided by other manufacturers instead.

The study has a number of limitations. First, the scarce availability of samples positive for some of the pathogens covered by the Allplex assays made deviations from the recommended evaluation strategy necessary for various parameters. Second, microscopical and culture results were not consistently available for the samples to be of use for the definitions of the positive and negative samples. Therefore, it cannot be completely excluded that apparently lacking sensitivity of the assessed Allplex assays may, at least in part, have been due to lacking specificity of the reference PCRs, although the latter can be considered as well-evaluated. Third, residual samples of different ages were included, so DNA degradation might have occurred in spite of appropriate sample storage deep-frozen at −80 °C. This limitation was attempted to be reduced by performing all PCRs from this study within a relatively short period of time of a few weeks, so all assays were run under comparable conditions. Fourth, lacking information on the clinical situation of the patients from which the samples were taken, in line with the ethical requirements of the study, prevented the use of such information for the evaluation of the test results regarding their clinical plausibility. Fifth, financial limitations made the hypothetical option of sequencing all the amplicons in order to support the decision on specific and non-specific PCR results unfeasible. Sixth, and also due to financial restrictions, the study could not be repeated by using the Allplex assays as the reference standard and the in-house PCR assays as competitors. If the apparent sensitivity limitations of the Allplex assays were mainly due to imperfect concordance, as suggested by previous assessments [9,10], similarly poor sensitivity results would have resulted for the in-house assays in case of such an inverted study design.

## 5. Conclusions

In spite of the above-mentioned limitations, it was shown that the evaluated Allplex assays provide good specificity, as well as inter- and intra-assay reproducibility, while the sensitivity still leaves room for improvement compared to the applied in-house competitor PCR assays. The manufacturer-recommended mode of nucleic acid extraction may partly account for the reduced sensitivity, but it is unlikely to be the only influence. Considering the fact that most of the assessed pathogens are facultatively pathogenic and may occur as colonizers without clinical symptoms as well, at least in high-endemicity settings, future studies should assess whether the assays’ sensitivity might nevertheless be sufficient for the detection of clinically relevant infections, which have been inconsistently reported to be associated with higher pathogen loads [47,48,49]. If even traces of the pathogen nucleic acids are detected, it can be difficult to discriminate infections from harmless colonization with enteropathogenic microorganisms, a problem that has been reported to be frequent in resource-poor tropical settings [50,51].

## Figures and Tables

**Table 1:** Results of the assessments with strongly positive samples. Calculated sensitivity values refer to the applied reference standards as described in the Methods section.

Target Pathogen	Sensitivity, n/n (%)	Cycle Threshold Values of the In-House PCR, Mean (Standard Deviation)	Cycle Thresho ld Values of the Allplex Assay, Mean (Standard Deviation)	Proportion of Native Stool Samples, n/n (%)	Proportion of Manual Nucleic Acid Extractions, n/n (%)	Proportions of Spiked Stool Samples, n/n (%)
Enteroaggregative *Escherichia coli* (EAEC)	10/15 (67%)	23.0 (±4.7)	33.3 (±5.0)	15/15 (100%)	n.a.	n.a.
Enteropathogenic *Escherichia coli* (EPEC)	8/15 (53%)	23.4 (±4.5)	35.5 (±3.6)	15/15 (100%)	n.a.	n.a.
*Escherichia coli* O157	9/9 (100%)	n.a.	33.0 ( ± 3.2)	9/9 (100%)	n.a.	n.a.
Enterotoxigenic *Escherichia coli* (ETEC)	12/15 (80%)	22.7 (±3.6)	32.5 (±4.8)	15/15 (100%)	n.a.	n.a.
Shiga toxin-producing *Escherichia coli* (STEC)	14/15 (93%)	n.a.	33.1 (±2.8)	15/15 (100%)	n.a.	n.a.
*Campylobacter* spp.	13/15 (87%)	24.5 (±3.8)	37.5 (±2.6)	15/15 (100%)	n.a.	n.a.
*Clostridioides difficile* toxin B	8/8 (100%)	n.a.	30.8 (±2.5	8/8 (100%)	n.a.	n.a.
*Salmonella* spp.	2/15 (13%)	26.1 (±3.8)	39.9 (±0.1)	15/15 (100%)	n.a.	n.a.
*Shigella* spp./enteroinvasive *Escherichia coli* (EIEC)	11/15 (73%)	22.3 (±4.5)	39.4 (±3.8)	15/15 (100%)	n.a.	n.a.
*Vibrio* spp. *	6/15 (40%)	n.a.	25.0 (±1.8)	n.a.	n.a.	15/15 (100%)
*Yersinia enterocolitica ^#^*	9/9 (100%)	20.8 (±4.3)	25.9 (±3.4)	n.a.	9/9 (100%)	n.a.
*Giardia duodenalis*	11/14 (79%)	22.5 (±4.6)	29.7 (±3.4)	14/14 (100%)	n.a.	n.a.
*Cryptosporidium* spp.	9/15 (60%)	26.2 (±3.1)	33.9 (±3.4)	15/15 (100%)	n.a.	n.a.
*Blastocystis hominis*	15/15 (100%)	n.a.	28.4 (±2.4)	15/15 (100%)	n.a.	n.a.
*Cyclospora cayetanensis*	0/15 (0%)	28.6 (±1.9)	n.e.	15/15 (100%)	n.a.	n.a.
*Entamoeba histolytica*	2/14 (14%)	29.4 (±7.2)	33.0 (±3.4)	14/14 (100%)	n.a.	n.a.
*Dientamoeba fragilis*	15/15 (100%)	23.2 (±4.1)	34.9 (±4.9)	15/15 (100%)	n.a.	n.a.
*Ancylostoma* spp.	2/2 (100%)	29.5 (±0.5)	32.6 (±0.9)	n.a.	2/2 (100%)	n.a.
*Ascaris* spp.	5/15 (33%)	29.3 (±1.6)	40.4 (±1.8)	15/15 (100%)	n.a.	n.a.
*Enterobius vermicularis*	1/6 (17%)	27.6 (±2.4)	33.7 (n.e.)	5/6 (83%)	1/6 (17%)	n.a.
*Enterocytozoon* spp./ *Encephalitozoon* spp.	10/15 (67%)	24.3 (±5.5)	30.7 (±4.8)	15/15 (100%)	n.a.	n.a.
*Hymenolepis* spp.	9/15 (60%)	25.1 (±3.0)	31.8 (±5.7)	11/15 (73%)	4/15 (27%)	n.a.
*Necator americanus*	3/15 (20%)	31.2 (±2.2)	36.5 (±3.0)	15/15 (100%)	n.a.	n.a.
*Strongyloides* spp.	1/15 (7%)	28.2 (±3.1)	37.7 (n.e.)	15/15 (100%)	n.a.	n.a.
*Taenia* spp.	4/15 (27%)	30.1 (±2.2)	38.0 (±1.3)	15/15 (100%)	n.a.	n.a.
*Trichuris trichiura*	1/15 (7%)	27.2 (±2.0)	37.2 (n.e.)	15/15 (100%)	n.a.	n.a.

PCRs for hypervirulent *C. difficile* and *Aeromonas* spp. are not included in the table, because the evaluations for these parameters were just based on stool material spiked with a dilution series of single strains. n.a. = not applicable. n.e. = not estimable. * Positively tested samples comprised 3x *V. cholerae* (comprising 2 out of 4 spiking strains tested), 1x *V. alginolyticus*, 1x *V. parahaemolyticus* and 1x *V. vulnificus*, while *V. fluvialis*, *V. furnissii* and *V. mimicus* were missed. ^#^ Of note, one included sample positive for *Yersinia pseudotuberculosis* was negative, confirming the assay’s specificity for *Y. enterocolitica*.

**Table 2:** Results of the assessments with weakly positive samples. Calculated sensitivity values refer to the applied reference standards as described in the Methods section.

Target Pathogen	Sensitivity, n/n (%)	Cycle Threshold Values of the In-House PCR, Mean (Standard Deviation)	Cycle Threshold Values of the Allplex Assay, Mean (Standard Deviation)	Proportion of Native Stool Samples, n/n (%)	Proportion of Manual Nucleic Acid Extractions, n/n (%)	Proportions of Spiked Stool Samples, n/n (%)
Enteroaggregative *Escherichia coli* (EAEC)	3/15 (20%)	32.9 (±1.8)	37.1 (±2.7)	15/15 (100%)	n.a.	n.a.
Enteropathogenic *Escherichia coli* (EPEC)	2/15 (13%)	33.1 (±2.0)	38.1 (±0.9)	15/15 (100%)	n.a.	n.a.
*Escherichia coli* O157	5/5 (100%)	n.a.	34.9 (±0.9)	12/12 (100%)	n.a.	n.a.
Enterotoxigenic *Escherichia coli* (ETEC)	2/15 (13%)	34.0 (±1.7)	37.5 (±0.6)	15/15 (100%)	n.a.	n.a.
Shiga toxin-producing *Escherichia coli* (STEC)	12/12 (100%)	n.a.	36.6 (±2.2)	5/5 (100%)	n.a.	n.a.
*Campylobacter* spp.	7/15 (47%)	32.7 (±1.6)	39.5 (±1.8)	15/15 (100%)	n.a.	n.a.
*Clostridioides difficile* toxin B	10/10 (100%)	n.a.	33.8 (±2.5)	10/10 (100%)	n.a.	n.a.
*Salmonella* spp.	0/15 (0%)	32.8 (±1.3)	n.e.	15/15 (100%)	n.a.	n.a.
*Shigella* spp./enteroinvasive *Escherichia coli* (EIEC)	1/15 (7%)	33.2 (±1.7)	38.8 (n.e.)	15/15 (100%)	n.a.	n.a.
*Vibrio* spp. *	7/15 (47%)	n.a.	29.1 (±2.1)	n.a.	n.a.	15/15 (100%)
*Yersinia enterocolitica*	3/3 (100%)	33.7 (±3.5)	24.4 (±0.5)	n.a.	3/3 (100%)	n.a.
*Giardia duodenalis*	0/15 (0%)	32.8 (±1.2)	n.e.	15/15 (100%)	n.a.	n.a.
*Cryptosporidium* spp.	2/15 (13%)	34.6 (±1.8)	41.1 (±0.4)	15/15 (100%)	n.a.	n.a.
*Cyclospora cayetanensis*	0/15 (0%)	35.6 (±2.4)	n.e.	15/15 (100%)	n.a.	n.a.
*Entamoeba histolytica*	0/15 (0%)	40.0 (±2.0)	n.e.	15/15 (100%)	n.a.	n.a.
*Dientamoeba fragilis*	6/15 (40%)	34.2 (±2.9)	40.4 (±1.3)	15/15 (100%)	n.a.	n.a.
*Ascaris* spp.	0/15 (0%)	34.7 (±1.7)	n.e.	15/15 (100%)	n.a.	n.a.
*Enterobius vermicularis*	0/8 (0%)	33.6 (±1.9)	n.e.	4/8 (50%)	4/8 (50%)	n.a.
*Enterocytozoon* spp./*Encephalitozoon* spp.	0/15 (0%)	32.0 (±1.2)	n.e.	15/15 (100%)	n.a.	n.a.
*Hymenolepis* spp.	8/15 (53%)	31.99 (±1.19)	38.0 (±1.7)	6/15 (40%)	9/15 (60%)	n.a.
*Necator americanus*	0/15 (0%)	36.6 (±1.6)	n.e.	15/15 (100%)	n.a.	n.a.
*Strongyloides* spp.	0/15 (0%)	36.6 (±1.3)	n.e.	15/15 (100%)	n.a.	n.a.
*Taenia* spp.	0/15 (0%)	37.5 (±2.3)	n.e.	15/15 (100%)	n.a.	n.a.
*Trichuris trichiura*	1/15 (7%)	32.4 (±1.3)	41.0 (n.e.)	15/15 (100%)	n.a.	n.a.

The PCRs for hypervirulent *C. difficile* and *Aeromonas* spp. are not included in the table, because the evaluation of these parameters was just based on stool material spiked with a dilution series of single strains. The PCR for *Blastocystis hominis* is not included, because all assessed microscopy-positive samples were considered as strongly positive by definition. The PCR for *Ancylostoma* spp. is not included, because no weakly positive samples were available for the assessment. n.a. = not applicable. n.e. = not estimable. * Positively tested samples comprised 4x *V. cholerae* (comprising 3 out of 4 spiking strains tested), 1x *V. alginolyticus*, 1x *V. parahaemolyticus* and 1x *V. vulnificus*, while *V. fluvialis*, *V. furnissii* and *V. mimicus* were missed.

**Table 3:** Intra- and inter-assay variations assessed with the same sample per parameter of the Allplex assays. For target parameters indicated by an asterisk (*), insufficient residual stool volumes of positive samples were available, so residual DNA from previous nucleic acid extractions had to be used for the assessments.

Target Parameter	Inter-Assay Variation as Variance of the Recorded Ct Values, in Brackets: Mean Ct value ± Standard Deviation (SD)	Intra-Assay Variation as Variance of the Recorded Ct Values, in Brackets: Mean Ct Value ± Standard Deviation (SD)
Enteroaggregative *Escherichia coli* (EAEC)	0.01 (24.8 ± 0.1)	0.01 (24.7 ± 0.1)
Enteropathogenic *Escherichia coli* (EPEC)	0.01 (29.0 ± 0.1)	0.09 (29.1 ± 0.3)
*Escherichia coli* O157	0.01 (23.9 ± 0.1)	0.09 (24.1 ± 0.3)
Enterotoxigenic *Escherichia coli* (ETEC)	0.16 (22.7 ± 0.4)	0.00 (22.2 ± 0.0)
Hypervirulent *Clostridioides difficile*	0.04 (27.1 ± 0.2)	0.09 (26.7 ± 0.3)
Shiga toxin-producing *Escherichia coli* (STEC)	0.00 (22.9 ± 0.0)	0.09 (22.9 ± 0.3)
*Aeromonas* spp.	0.04 (26.9 ± 0.2)	0.04 (27.0 ± 0.2)
*Campylobacter* spp.	0.04 (36.4 ± 0.2)	0.16 (36.6 ± 0.4)
*Clostridioides difficile* toxin B	0.04 (23.8 ± 0.2)	0.04 (23.8 ± 0.2)
*Salmonella* spp.	0.16 (41.1 ± 0.4)	0.16 (41.2 ± 0.4)
*Shigella* spp./ enteroinvasive *Escherichia coli* (EIEC)	0.04 (33.0 ± 0.2)	0.04 (33.0 ± 0.2)
*Vibrio* spp.	0.09 (24.2 ± 0.3)	0.01 (24.6 ± 0.1)
*Yersinia enterocolitica* *	0.09 (26.4 ± 0.3)	0.64 (25.0 ± 0.8)
*Giardia duodenalis*	0.04 (24.2 ± 0.2)	0.16 (24.1 ± 0.4)
*Cryptosporidium* spp.	0.01 (26.4 ± 0.1)	0.01 (26.5 ± 0.1)
*Blastocystis hominis* *	0.09 (25.2 ± 0.3)	0.01 (25.3 ± 0.1)
*Cyclospora cayetanensis* *	0.64 (36.6 ± 0.8)	0.09 (37.2 ± 0.3)
*Entamoeba histolytica*	0.01 (35.9 ± 0.1)	0.16 (36.1 ± 0.4)
*Dientamoeba fragilis* *	0.25 (23.2 ± 0.5)	0.01 (23.1 ± 0.1)
*Ancylostoma* spp. *	0.09 (32.0 ± 0.3)	0.09 (32.4 ± 0.3)
*Ascaris* spp. *	0.16 (28.0 ± 0.4)	0.04 (28.3 ± 0.2)
*Enterobius vermicularis* *	0.01 (32.0 ± 0.1)	0.16 (32.1 ± 0.4)
*Enterocytozoon* spp./ *Encephalitozoon* spp.	0.01 (21.5 ± 0.1)	0.04 (21.6 ± 0.2)
*Hymenolepis* spp. *	0.01 (29.8 ± 0.1)	0.01 (29.8 ± 0.1)
*Necator americanus* *	0.04 (29.8 ± 0.2)	0.00 (29.9 ± 0.0)
*Strongyloides* spp.	7.29 (38.5 ± 2.7)	0.16 (36.3 ± 0.4)
*Taenia* spp. *	0.36 (36.6 ± 0.6)	0.36 (37.4 ± 0.6)
*Trichuris trichiura* *	0.16 (31.4 ± 0.4)	0.04 (31.6 ± 0.2)

Ct = cycle threshold; SD = standard deviation. * Assessments performed with previous nucleic acid extractions only, because the remaining native stool volumes were insufficient.

**Table 4:** Comparison of the Ct values with the Allplex assays after automated and manual nucleic acid extraction. Available Ct values from the in-house PCRs have been prefixed as external references. Varying denominators arose from the differing availabilities of the residual sample volumes.

PCR Target Species	External Reference: Number and Proportion of Positives (n/n, %) and Ct-Values Measured with the in-House PCR after Manual Nucleic Acid Extraction, Mean (Standard Deviation)	Number and Proportions of Positives (n/n, %) And Ct-Values Measured with the Allplex Assay after Automated Nucleic Acid Extraction, Mean (Standard Deviation)	Number and Proportion of Positives (n/n, %) And Ct-Values Measured with the Allplex Assay after Manual Nucleic Acid Extraction, Mean (Standard Deviation)	Significance Level (P) by Mann Whitney U-Testing of Paired Samples for Differences between Ct-Values Measured with the Allplex Assay after Automated and after Manual Nucleic Acid Extraction for Samples Positive in Both Approaches; In Brackets: Number of Assessed Ct-Value Pairs
Enteroaggregative *Escherichia coli* (EAEC)	30/30, 100%; 28.0 ± 6.1	13/30, 43,3%; 34.2 ± 4.9	19/30, 63.3%, 34.9 ± 4.7	0.048 (n = 13)
Enteropathogenic *Escherichia coli* (EPEC)	30/30, 100%; 28.2 ± 6.0	10/30, 33.3%; 36.0 ± 3.3	13/30, 43.3%; 35.5 ± 3.6	0.027 (n = 10)
*Escherichia coli* O157	n.a.	14/14, 100%, 33.7 ± 2.8	10/14, 71.4% 37.2 ± 3.0	0.002 (n = 10)
Enterotoxigenic *Escherichia coli* (ETEC)	30/30, 100%; 28.3 ± 6.3	14/30, 46.7%; 33.2 ± 4.8	16/30, 53,3%; 32.9 ± 4.8	0.151 (n = 13)
Shiga toxin-producing *Escherichia coli* (STEC)	n.a.	26/27, 96.3%; 34.7 ± 3.1	18/27, 66.7%; 38.0 ± 2.8	<0.0001 (n = 18)
*Campylobacter* spp.	30/30, 100%; 28.6 ± 5.1	20/30, 66.7%; 38.2 ± 2.6	20/30, 66.7%; 34.3 ± 3.2	<0.0001 (n = 19)
*Clostridioides difficile* toxin B	n.a.	18/18, 100%; 32.5 ± 2.9	18/18, 100%; 36.2 ± 2.3	<0.0001 (n = 18)
*Salmonella* spp.	30/30, 100%; 29.4 ± 4.4	2/30, 6.7%; 39.9 ± 0.1	3/30, 10%; 40.6 ± 1.6	n.e. (n = 2)
*Shigella* spp./enteroinvasive *Escherichia coli* (EIEC)	30/30, 100%; 27.8 ± 6.4	12/30, 40%; 39.3 ± 3.7	18/30, 60%; 39.2 ± 3.2	0.520 (n = 11)
*Giardia duodenalis*	29/29, 100%; 27.8 ± 6.1	11/29, 37.9%; 29.7 ± 3.4	12/29, 41.4%; 28.6 ± 4.2	0.042 (n = 11)
*Cryptosporidium* spp.	30/30, 100%; 30.4 ± 4.9	11/30, 36.7%; 35.2 ± 4.1	14/30, 46.7%; 34.9 ± 4.7	0.010 (n = 11)
*Blastocystis hominis*	n.a.	15/15, 100%; 28.4 ± 2.4	15/15, 100%; 28.4 ± 2.2	0.934 (n = 15)
*Cyclospora cayetanensis*	30/30, 100%; 32.1 ± 4.1	0/30, 0%; n.e.	2/30, 6.7%; 38.8 ± 1.2	n.e. (n = 0)
*Entamoeba histolytica*	29/29, 100%; 34.9 ± 7.5	2/29, 6.9%; 33.0 ± 3.4	3, 10.3%; 32.6 ± 6.2	n.e. (n = 2)
*Dientamoeba fragilis*	30/30, 100%; 28.7 ± 6.5	21/30, 70%; 36.5 ± 4.9	23/30, 76.7%; 34.1 ± 5.5	0.005 (n = 19)
*Ascaris* spp.	30/30, 100%; 32.0 ± 3.2	5/30, 16.7%; 40.4 ± 1.8	27/30, 90%; 35.4 ± 3.1	0.063 (n = 5)
*Enterobius vermicularis*	14/14, 100%; 31.1 ± 3.6	0/9, 0%; n.e.	3/14, 21.4%; 35.3 ± 3.0	n.e. (n = 0)
*Enterocytozoon* spp./ *Encephalitozoon* spp.	30/30, 100%; 28.1 ± 5.5	10/30, 33.3%; 30.7 ± 4.8	11/29, 37.9%; 29.9 ± 5.5	0.074 (n = 9)
*Hymenolepis* spp.	30/30, 100%; 28.5 ± 4.1	5/17, 29.4%; 35.9 ± 2.8	21/30, 70%; 34.5 ± 4.9	0.063 (n = 5)
*Necator americanus*	30/30, 100%; 33.9 ± 3.3	3/30, 10%; 36.5 ± 3.0	21/30, 70%; 37.2 ± 3.6	0.250 (n = 3)
*Strongyloides* spp.	30/30, 100%; 32.4 ± 4.8	1/30, 3.3%; 37.6 ± 0	4/30, 13.3%; 34.9 ± 2.7	n.e. (n = 1)
*Taenia* spp.	30/30, 100%; 33.8 ± 4.3	4/30, 13.3%; 38.0 ± 1.3	7/29, 24.1%; 37.2 ± 1.5	0.125 (n = 4)
*Trichuris trichiura*	30/30, 100%; 29.8 ± 3.1	2/30, 6.7%; 39.1 ± 1.9	28/30, 93.3%; 36.3 ± 2.9	n.e. (n = 2)

Spiked samples were excluded from the assessment, so the parameters *Vibrio* spp., hypervirulent *C. difficile* and *Aeromonas* spp. are not present. *Yersinia enterocolitica* and *Ancylostoma* spp. are not included, because no residual stool material from the positive samples was available for a test comparison. n.a. = not applicable. n.e. = not estimable.

## Data Availability

All relevant data have been presented in the text. Raw data can be made available upon reasonable request.
